# Strategies for High-Performance Resource-Efficient Compression of Neural Spike Recordings

**DOI:** 10.1371/journal.pone.0093779

**Published:** 2014-04-11

**Authors:** Palmi Thor Thorbergsson, Martin Garwicz, Jens Schouenborg, Anders J. Johansson

**Affiliations:** 1 Department of Experimental Medical Science, Lund University, Lund, Sweden; 2 Department of Electrical and Information Technology, Lund University, Lund, Sweden; 3 Neuronano Research Center, Lund University, Lund, Sweden; McGill University, Canada

## Abstract

Brain-machine interfaces (BMIs) based on extracellular recordings with microelectrodes provide means of observing the activities of neurons that orchestrate fundamental brain function, and are therefore powerful tools for exploring the function of the brain. Due to physical restrictions and risks for post-surgical complications, wired BMIs are not suitable for long-term studies in freely behaving animals. Wireless BMIs ideally solve these problems, but they call for low-complexity techniques for data compression that ensure maximum utilization of the wireless link and energy resources, as well as minimum heat dissipation in the surrounding tissues. In this paper, we analyze the performances of various system architectures that involve spike detection, spike alignment and spike compression. Performance is analyzed in terms of spike reconstruction and spike sorting performance after wireless transmission of the compressed spike waveforms. Compression is performed with transform coding, using five different compression bases, one of which we pay special attention to. That basis is a fixed basis derived, by singular value decomposition, from a large assembly of experimentally obtained spike waveforms, and therefore represents a generic basis specially suitable for compressing spike waveforms. Our results show that a compression factor of 99.8%, compared to transmitting the raw acquired data, can be achieved using the fixed generic compression basis without compromising performance in spike reconstruction and spike sorting. Besides illustrating the relative performances of various system architectures and compression bases, our findings show that compression of spikes with a fixed generic compression basis derived from spike data provides better performance than compression with downsampling or the Haar basis, given that no optimization procedures are implemented for compression coefficients, and the performance is similar to that obtained when the optimal SVD based basis is used.

## Introduction

Brain-machine interfaces (BMIs) are systems that provide a signal pathway between the central nervous system (CNS) and the outside world and thereby a means of observing the neuronal activity that underlies behavior. One class of BMIs employ intracranially implanted microelectrodes to record the changes in extracellular potential induced by activities of neurons surrounding them [1]. The extracellular recording is composed of spikes representing action potentials in near-by neurons, noise consisting of spikes from distant neurons, local field potentials (LFPs) representing synaptic activity and thermal noise generated in the recording electronics [2].

By isolating the spiking components of the individual neurons that contribute to the extracellular recording, the firing patterns of those neurons can be characterized and correlated with events or learning processes in the motor or sensory domains [3] to reveal the dynamics of neuronal circuits that govern behavior. The fundamental steps in this procedure are (a) spike detection and extraction, (b) spike alignment and (c) spike sorting. Spike detection is commonly based on detecting the local increase in signal energy or amplitude associated with the firing of a spike and its aim is to pinpoint the temporal occurrence of spike waveforms. Spike alignment is important for the subsequent spike sorting step and it involves shifting the detected spike waveforms in time to have them aligned with respect to a given waveform landmark, e.g. the point of maximum amplitude. Spike sorting typically involves first extracting spike features (i.e. waveform characteristics that ideally are the same for spikes coming from the same neuron but different for spikes coming from different neurons) and then classifying the spikes based on the extracted features [4].

BMIs are often implemented with multiple recording channels (multielectrode arrays), which results in the recording and processing of vast amounts of data [5]. In [6], it was shown that to ensure sustained performance in spike detection and spike sorting, the extracellular signal needed to be acquired at a sampling rate of 25 kHz and an effective sampling resolution of at least 9 bits. Assuming a sampling resolution of 10 bits, this results in a raw data rate of 250 kbps per channel. Thus, for a state-of-the-art electrode array with 100 channels [7], the total data rate becomes 25 Mbps, which typically requires a physical (wired) link between the implanted and external parts of the BMI.

Although sufficient with regard to data transmission, wired BMIs are associated with risks of post-surgical complications due to transcutaneous leads. They also strongly limit subject mobility. These drawbacks of wired BMIs make them unpractical for chronic applications where the behavior of the subject is observed under unrestrained conditions. Wireless BMIs ideally solve the problems that have to do with the physical link, but they are limited in terms of energy resources and wireless link capacity, and the data processing and RF transmitter operation can potentially give rise to harmful heat dissipation in tissues surrounding the implant. All of those are challenges whose significance increases with an increased number of neural recording channels.

In order to overcome the challenges associated with multi-channel wireless BMIs, low-complexity data compression is needed. Compression removes redundant information from the recorded signal and thereby the data rate is effectively lowered, which leads to better utilization of the wireless link and thereby also minimizes the energy used to transmit each bit of information. Low computational complexity further decreases the energy consumption and heat dissipation, and it is expected to increase the operational stability, which is vital to the success of wireless BMIs.

A simple form of compression involves detection and extraction of spikes, resulting in the transmission of timestamps and spike waveforms. At a sampling rate of 25 kHz, a sampling resolution of 10 bits and a spike duration of 2.5 ms, a data rate of 640 bits per spike is achieved (excluding the timestamps). However, it has been shown that there is significant redundancy involved in transmitting the entire spike waveform. By linear transformation with a suitable compression basis, the extracted spike can be transform coded and described by a small set of transform (compression) coefficients instead of the full set of waveform samples [8]–[10]. For instance, if the spike is adequately approximated by the linear combination of eight compression basis waveforms, the compression coefficients can be taken as the transform coefficients corresponding to those eight basis waveforms and the data rate is further decreased to 80 bits per spike. The adequacy of the approximation refers to how well the spikes, after transmission and reconstruction, correspond to the original uncompressed spikes and how the performance in the subsequent analysis, i.e. spike sorting, is influenced by the compression.

The choices of compression basis and compression coefficients to use in transform coding are of key importance to the performance of the wireless BMI. Ideally, the compression basis is derived from the data that is to be compressed by means of, for instance singular value decomposition (SVD), where an orthonormal basis is found and the transform coefficients are ordered by significance, making the selection of compression coefficients straightforward (the first 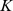 coefficients are chosen). However, finding and maintaining such an optimal basis is computationally demanding and thus not feasible in a low-power wireless implant. A more feasible solution is to employ a fixed compression basis that does not need to be adapted to the data each time, and therefore reduces the computational complexity significantly. Generic bases, such as various wavelet bases and the Walsh-Hadamard basis, have previously been used for this purpose, but since they are generally not derived from spike data and are not ordered according to expected transform coefficient significance, they suffer from both suboptimal degree of compression as well as the need for selecting which transform coefficients to transmit in each case [11]–[14].

The aim of the present paper was therefore to analyze how the choice of compression basis, in combination with a spike detector and system architecture (a given distribution of processing tasks among the parts of the wireless BMI), influences the accuracy in spike reconstruction and spike sorting when compressing and processing synthetic extracellular recordings with realistic a priori known properties. Synthetic recordings were used in order to facilitate quantitative analysis of performance in spike sorting. First, all configurations are analyzed and compared in order to provide general insight into their relative performances and dependencies of simulation variables. Second, we focus on two configurations in which good performance is obtained in the first part. These cases are of special interest since they involve a compression basis that is derived by SVD from a large set of experimentally obtained spike waveforms in the cat cerebellum [15], [16]. Since the basis is derived from actual spike data, it is expected to provide a high degree of compression, and since it is derived by SVD, the transform coefficients are expected to be ordered according to significance, making the selection of compression coefficients straightforward. These features are attractive from the points of view of utilizing the limited wireless link capacity as well as minimizing energy consumption and heat dissipation.

## Methods

### 0.1 Synthetic Test Data

**Figure 1:**
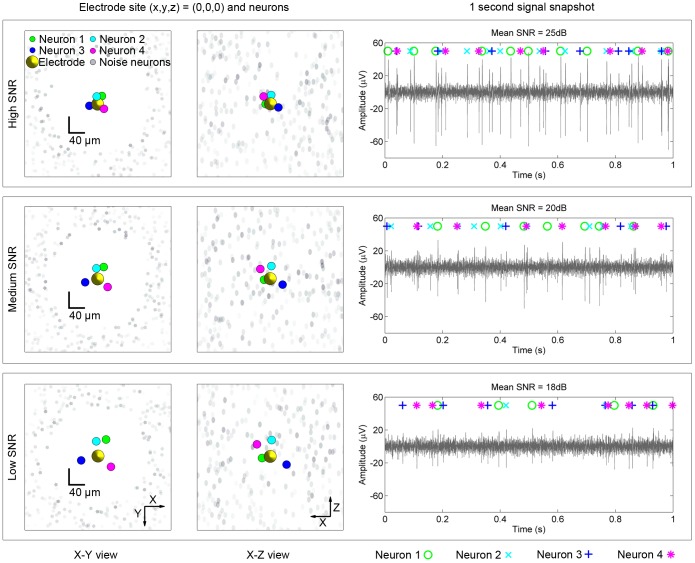
One of nineteen electrode sites and a snapshot of the corresponding signal from each of the multi-channel test recordings (high, medium and low). The SNR was varied by altering the placements of neurons 1 to 4 away from the linear electrode array placed along the 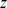 axis while the hollow cylindrical volume containing noise neurons was kept fixed. The placements of the neurons were altered by multiplying their Cartesian coordinates for the high SNR case by factors of 1.5 and 2 for medium and low SNR respectively. The left part of the figure shows the placements of the target neurons (colored dots around origin), noise neurons (gray dots far away from origin) and the electrode site 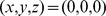 (yellow dot in origin). The sizes of the indicators do not reflect the true sizes of the neurons and the electrode, but only their positions. Only the X-Y and X-Z views are shown. Note that each test recording contained eighteen additional electrode sites, arranged along the 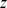-axis. The right part of the figure shows one second of the total of five minutes of recording with the true spike identities labeled and color coded.

The simulator described in [17] was used here to synthesize three nineteen-channel test recordings with varying signal-to-noise ratios (high, medium and low SNR). The recordings were five minutes long. In all three recordings, a linear array of nineteen evenly spaced electrodes was placed along the 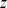-axis (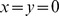, 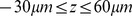, 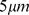 spacing). Noise neurons were placed at random positions (density of 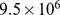 neurons/cm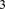 [18]) within a hollow cylinder concentric with the 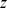-axis, as shown in Figure 1. The inner and outer boundaries of the hollow cylindrical volume were at 120 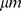 and 250 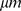 from the 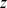-axis respectively and its floor and ceiling were at 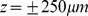 respectively.

Four target neurons (one of each neuron model derived in [17]) were placed inside the hollow space of the noise neuron cylinder. For the high SNR recording, the neurons were placed at positions of (10,20,−2)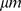, (−2,18,20)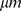, (−20 −5 −10)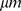 and (16,−13,15)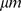. For the medium and low SNR recordings, these coordinates were multiplied by factors of 1.5 and 2 respectively, i.e. moving each neuron along a linear path from the origin. Moving the neurons away from the electrode array resulted in decreasing their spike amplitudes and thus decreasing the SNR since the noise neuron cylinder was not altered. The multiplication factors were chosen empirically to provide clearly varying SNRs and resulted in the neurons being at distances of approximately 20, 30 and 40 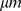 from the electrode array (in the 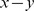 plane) for the high, medium and low SNR respectively. Assuming that spikes from neurons within a radius of 50 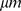 can be detected [19], these distances are reasonable.

Figure 1 illustrates the arrangement of neurons and the electrode site used in each case for the first part of the performance estimation, i.e. 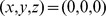 (see later section) as well as one second long segments of the signal in each SNR case. Note that each test recording contained eighteen electrode sites in addition to the one shown in Figure 1. Note also that the three test recordings were generated individually, meaning that the actual locations of noise neurons and actual spike times of all neurons varied between the recordings. However, the statistical properties used to generate locations and spike times did not vary between the recordings. The recordings were sampled at 25 kHz and bandpass filtered between 300 Hz and 5 kHz.

All neurons were assumed to have gamma distributed inter-spike intervals [20]. For each noise neuron, a random mean firing rate was chosen from a uniform distribution between 1 and 50 spikes/second. For each target neuron, a random mean firing rate was chosen from a uniform distribution between 1 and 10 spikes/second.

Signal to noise ratio was calculated in a similar manner as described in [21]. For a given recording and a given electrode site, we defined the SNR for the 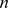-th neuron as 
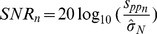
(1)where 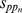 is the peak-to-peak amplitude of the mean spike waveform of the neuron measured at the electrode site and 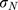 is the standard deviation of background noise estimated according to [22]

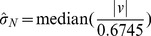
(2)where 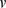 is the digitized signal. The mean SNR across the target neurons in each case is reported.

### 0.2 System Architectures

**Figure 2:**
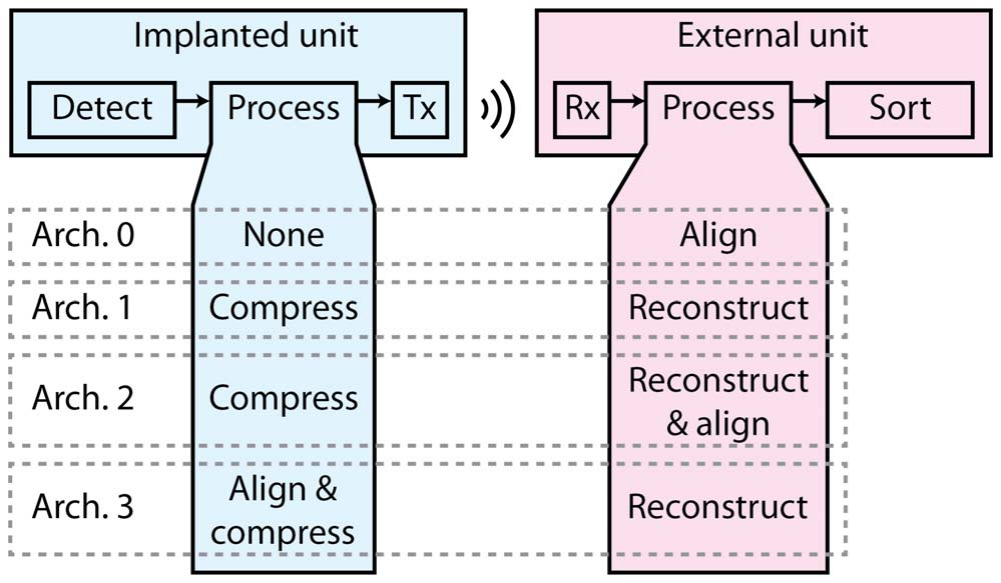
An illustration of the system architectures considered in the comparison. The ''Tx'' and ''Rx'' blocks refer to the wireless transmitter and receiver, respectively. See text for explanations.

Three different system architectures involving spike waveform compression were considered in our analysis (architectures 1 to 3 in Figure 2), in addition to a reference architecture in which no compression was performed (architecture 0). As illustrated in Figure 2, a given system architecture constitutes a specific combination and distribution of processing tasks among the parts of the wireless BMI. The main processing tasks were assumed to be the alignment, compression and reconstruction of detected spike waveforms. Spike detection was performed in the time domain in order to minimize the number of transform coding operations – a task that involves matrix multiplication and thus a significant increase in computational complexity if operated continuously. The processing steps are discussed in more detail in the following sections.

In architecture 0 (the reference architecture), the extracted spike waveforms were transmitted without compression and aligned in the external unit prior to spike sorting. In architecture 1, the extracted spike waveforms were compressed in the implanted unit. Architecture 2 was similar to architecture 1, but with an additional alignment step in the external unit prior to spike sorting. In architecture 3, the extracted spikes were aligned in the implanted unit prior to compression.

### 0.3 Spike Detection

**Figure 3:**
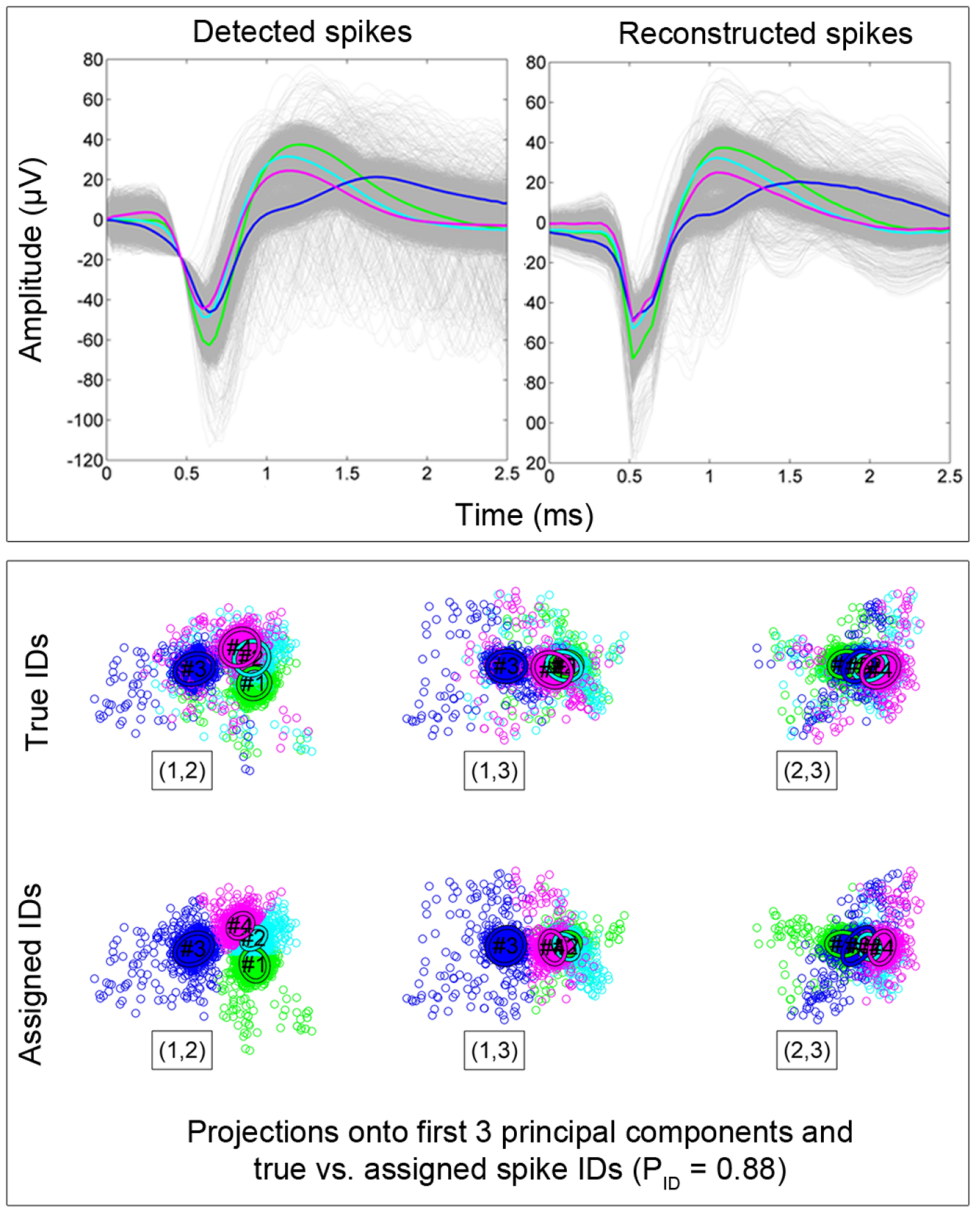
The upper panel shows detected and reconstructed spikes using the ABS detector and compression with the *fixed 2* basis (8 compression coefficients) and architecture 3 (high SNR, 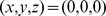). Mean spike waveforms are color coded according to their neurons of origin. The lower panel shows the projections of reconstructed spikes onto the first three principal components (marked (1,2), (1,3) and (2,3)). In the upper and lower rows, spikes in the PCA feature space are color coded according to their true and assigned identities, respectively. Clustering was performed with K-means. The overall spike sorting accuracy in this case was 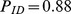.

We implemented *ABSolute value* (ABS) and *Nonlinear Energy Operator* (NEO) spike detection, both of which have been shown to provide a good combination of performance and computational complexity [6], [23], [24]. NEO has been shown to be more robust to background noise and provide spike detection jitter that is beneficial for spike sorting, but its computational complexity is significantly higher [6], [13], [23]. Spike detection jitter refers to the misalignment of extracted spike waveforms that arises when different spikes cross the detection threshold at different time instances within the waveform [2]. ABS is attractive due to its simplicity, but it requires an extra spike alignment step (see Section 0.4) due to the spike detection jitter it introduces (see Figure 3). Spike duration was assumed to be 2.5 milliseconds.

In order to avoid potential errors in the estimation of spike sorting accuracy caused by false positive spike detections, we used true spike times provided with the synthetic recordings to extract spike waveforms from the recordings and then introduced the spike detection jitter afterward. Detection thresholds (see Sections 0.3.1 and 0.3.2) were used to find the detection time (time instance of threshold crossing) for each extracted waveform that passed the threshold. Spikes that did not pass the threshold were discarded. We then used a discrete-time delay filter to shift each waveform in time to have the threshold crossing occur at the most frequent detection time across all spikes. Spikes whose threshold crossing time deviated by more than 1.5 standard deviations from the most frequent value were discarded as outliers. These cases usually represented overlapping spikes. Spike waveforms were upsampled to a sampling rate of 200 kHz before introducing the jitter and were then downsampled to 25 kHz again afterward. ABS and NEO spike detection are briefly described below.

#### 0.3.1 ABSolute Value Spike Detection

In ABS detection, a threshold of 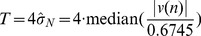
(3)where 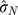 is the estimated standard deviation of background noise and 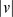 is the digitized signal's amplitude, is applied to the absolute value of the signal [22]. This is equivalent to simultaneously applying a positive and a negative threshold to the raw signal.

#### 0.3.2 Nonlinear Energy Operator Spike Detection

In NEO detection, a threshold 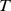 is applied to the nonlinear energy operator (NEO) 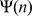 of the signal 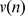. The nonlinear energy operator is given by 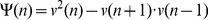
(4)and the threshold is taken as 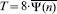
(5)where 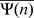 is the mean value of the NEO [23], [25].

### 0.4 Spike Alignment

For the architectures that involved a spike alignment step (architectures 0, 2 and 3), spikes were aligned at their point of maximum absolute amplitude within a time interval of approximately half a millisecond after the detection time. This was assumed to correspond to aligning the spikes on the maximum value of the detected peak or valley. We chose maximum amplitude alignment due to its simplicity, since it only involves finding the maximum absolute value of the signal within a short time window. More sophisticated approaches have been described, such as the center-of-mass alignment [2], which takes into account the entire waveform and is thus less sensitive to noise. Employing such a measure would have been feasible at the external unit (architectures 0 and 2), but not at the implanted unit (architecture 3). With that in mind, we selected the approach that was feasible with respect to the implanted unit and used it in all cases in order to allow a direct comparison.

For architectures 0 and 2 (spike alignment on the external unit), spikes were upsampled to a sampling rate of 200 kHz prior to alignment and were then downsampled back to 25 kHz after alignment. For architecture 3 (spike alignment on the implant), the alignment was performed at the initial sampling rate of 25 kHz in order to minimize the increase in computational complexity introduced by placing the alignment step in the implant. The alignment was performed using delay filters in the same way as when applying spike detection jitter (see Section 0.3).

### 0.5 Spike Compression and Reconstruction

Spike compression was performed by transforming detected spike waveforms with five different compression bases and a fixed number of compression coefficients were assumed to be transmitted and used for spike reconstruction. The transform was obtained as 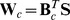
(6)where the 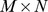 matrix 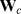 contains the full set of transform coefficients, the 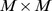 matrix 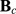 contains the 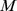 sample long basis waveforms of the compression basis in its columns and the 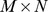 matrix 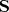 contains the 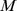 sample long spike waveforms in its columns. The fixed set of 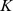 (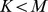) compression coefficients was extracted from 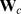 by introducing the 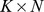 dimensionality reduction matrix 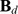. The structure of the dimensionality reduction matrix was chosen specifically for each compression basis to remove specific coefficients and it was introduced for the sake of generalizing the procedure for various compression bases. The 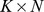 matrix of compression coefficients was then obtained as 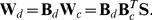
(7)The compression and dimensionality reduction bases were assumed to be known at the external unit and were used to reconstruct the spike waveforms according to 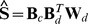
(8)where 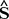 is the reconstructed spike matrix. For compression with the *downsampling* basis, the reconstruction involved an additional lowpass filtering step for interpolation (see Section 0.5.4).

**Figure 4:**
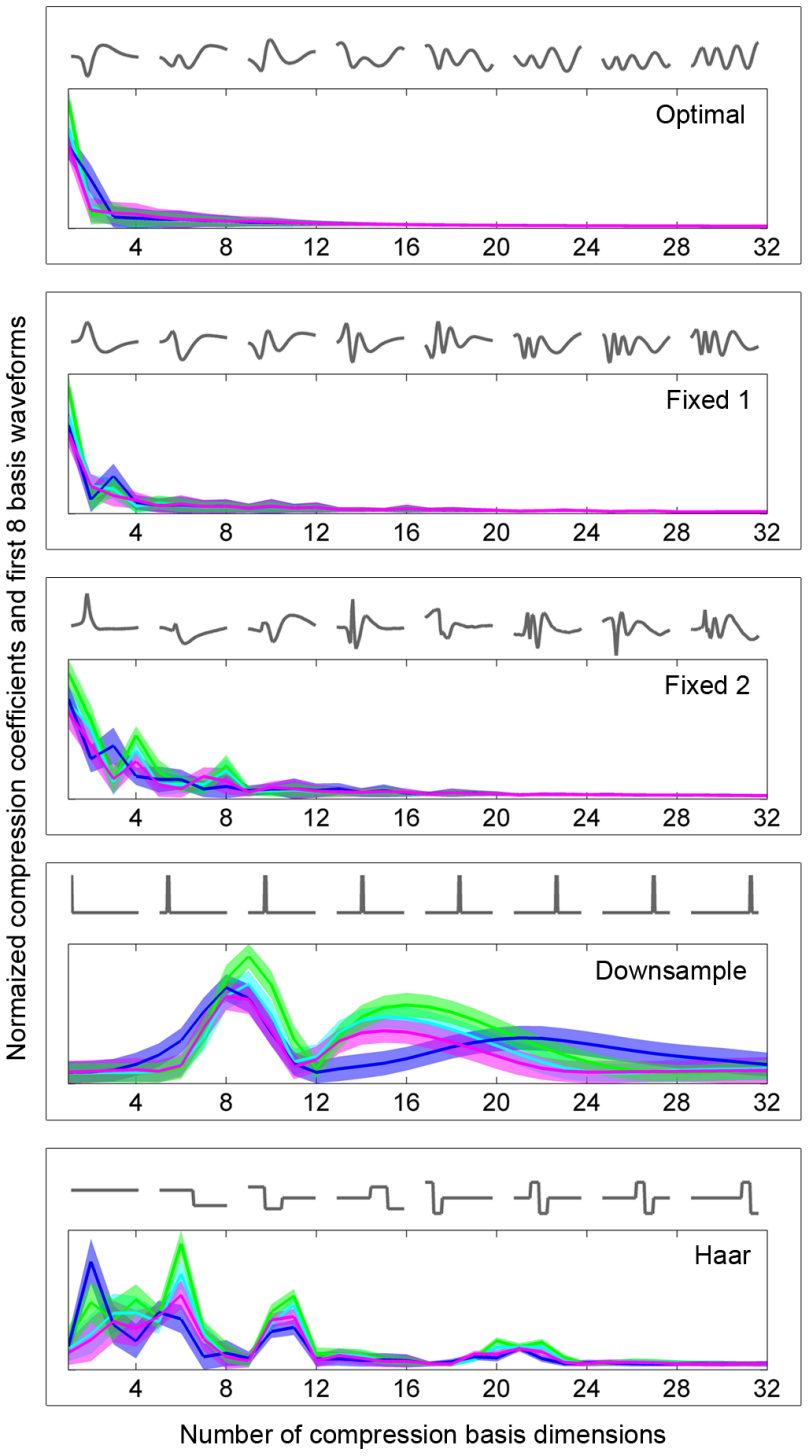
Eight basis waveforms from the five compression bases that were included (*optimal*, *fixed 1*, *fixed 2*, *downsample* and *Haar*) and absolute values of 32 compression coefficients of the spikes from the high SNR recording at electrode site 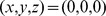. The coefficients are given as mean plus/minus one standard deviation for the spikes of each of the four target units (green, light blue, dark blue and pink). The coefficient spectra show the varying sparsification levels provided by the different bases, the *optimal* basis providing the highest sparsification and the *downsampling* basis the lowest.

The five compression bases that were included are briefly discussed below. Figure 4 shows the first eight basis waveforms of each basis and the absolute values of 32 compression coefficients in each basis for the high SNR recording at electrode site 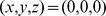. The distributions of transform coefficients show that the bases introduced various degrees of sparsity, indicating that they provided various degrees of compression. Apart from providing different levels of compression, the bases also provided different distributions of coefficients within the coefficient spectra. As touched upon in the introduction, the bases derived by SVD (*optimal*, *fixed 1* and *fixed 2*) all have the attractive property of providing coefficients that are concentrated at the lower end of the spectrum, making the selection of compression coefficients straightforward (the first 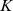 coefficients can be selected). This is in contrast to the *downsampling* and *Haar* bases, in whose coefficient spectra the first 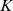 coefficients are not necessarily the most significant ones. Also, the SVD based bases generally tend to provide a greater degree of sparsification, indicating that they allow a higher degree of compression.

#### 0.5.1 Optimal Basis

This basis was found by performing singular value decomposition (SVD) on the matrix 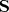 containing the detected spike waveforms in its columns. The *optimal* basis was included as a reference case since it involves basis waveforms that are derived directly from the waveforms that are to be compressed. The orthonormal basis waveforms were obtained as the columns of the 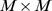 unitary matrix 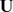 in the singular value decomposition 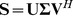
(9)where the diagonal elements of the 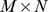 diagonal matrix 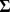 are the singular values (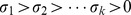, 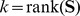) of 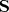 and the columns of the 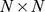 matrix 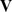 are the right-hand singular vectors of 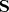 [26]. The basis vectors in the columns of 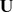 correspond to the descending singular values in 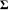 and are thus ordered in descending order according to the relative amount of spike-shape variance in 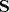 that they describe. Since the dimensions of the basis given by the SVD are arranged in this way, i.e. in decreasing order of significance, the dimensionality reduction matrix 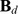 was taken as the first 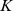 rows of the 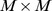 identity matrix (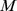 is the number of samples in each spike waveform, or the original dimensionality). As mentioned previously, this is an attractive property of SVD-based compression bases that results in most of the waveform information being concentrated in the lower range of the transform coefficients.

#### 0.5.2 Fixed Basis 1

This basis was found by performing SVD on a matrix containing 40.000 synthetic spike waveforms obtained by calculating the measured spike waveform in 10.000 random measurement points surrounding each of the four model neurons derived in [17]. This basis was assumed to represent the generic basis that was well tuned to the data, since it was derived from the same neuron models as the test data but not derived from the test data in each case. The dimensionality reduction matrix was the same as that for the *optimal* basis.

#### 0.5.3 Fixed Basis 2

This basis was obtained by performing SVD on the matrix of spike waveforms contained in the library of mean spike waveforms from recordings in the cat cerebellum used in [16]. Since this basis (obtained empirically) was entirely unrelated to the test data (derived from mathematical models of CA1 pyramidal neurons), it was assumed to be a truly generic fixed basis. Since this was an SVD based basis, the transform coefficients for spikes were expected to be mostly concentrated at the lower end of the spectrum (see Figure 4). Thus, the dimensionality reduction matrix was the same as that for the *optimal* basis. This basis was the most interesting one within the context of this paper, since it represents the generic compression basis where the derivation of the basis waveforms is entirely independent of the spike data that is to be compressed.

#### 0.5.4 Downsampling Basis

This basis was included as the simplest form of spike compression, namely that of discarding samples. The compression matrix 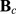 was taken as the 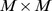 identity matrix and the dimensionality reduction matrix 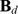 was obtained by removing all but every 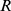-th row from the 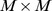 identity matrix where 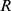 was the downsampling factor obtained by rounding the ratio 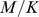 to the nearest integer. Having obtained the reconstructed spike matrix 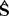 according to Eq. 8, the reconstructed waveforms were filtered in the frequency domain by a lowpass interpolation filter [27]. Note that since we wanted to examine the effects of simply discarding samples, no antialiasing filtering was applied prior to downsampling.

#### 0.5.5 Haar Wavelet Basis

This basis was obtained by constructing the 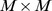 Haar matrix, whose columns contain the discrete time Haar basis waveforms. Although not necessarily optimal, the dimensionality reduction matrix was taken as the first 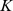 rows of the 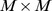 identity matrix. This choice was made since selecting the optimal compression coefficients would result in a need for implementing an optimization procedure on the implant, which would lead to a significant increase in complexity.

### 0.6 Spike Sorting

We performed feature extraction and clustering at the external unit with principal component analysis (PCA) and K-means respectively. In PCA, an ordered set of orthonormal basis waveforms is derived from the spike waveforms and the projections of the spikes onto the first 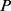 dimensions of this basis are used as features in spike sorting [4]. In K-means, data points are assigned to clusters that form gradually and ideally their means converge to the true cluster means [28]. We provided the true number of clusters (four neurons) as input to the K-means algorithm.

PCA is a widely used approach for feature extraction in spike sorting and has been shown to perform well in comparison to other feature extraction approaches, such as the discrete wavelet transform (DWT) and discrete derivatives (DD) [23]. For the DWT, this applies especially when the wavelet basis is badly tuned to the data [29] or when feature selection is not straightforward [6]. DD has been shown to provide similar performance as PCA, but as DWT, it requires a feature selection step [23]. We used the first three PCA weights as spike features. Since we assumed spike sorting to be performed at the external unit, the need for prioritizing computationally simple spike sorting algorithms was essentially eliminated.

### 0.7 Evaluation of Performance

System configurations (combination of a spike detector, system architecture and compression basis) were compared in terms of spike reconstruction accuracy and spike sorting accuracy. To quantify spike reconstruction accuracy, we used a waveform similarity measure similar to the one employed in [30]. The reconstruction accuracy was first calculated for each reconstructed spike as the maximum value of the cross-correlation function between the spike and the true mean spike for the neuron in question and the mean reconstruction accuracy across all spikes in a given recording, 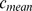, was reported. Overall spike sorting accuracy, 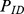, for a given case was estimated in the same way as described in [17], i.e. in terms of the total percentage of spikes that were identified correctly.

To be able to compare the relative overall performances of the system configurations, we introduced a score, ranging from zero to one, given by 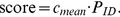
(10)The effective minimum number of compression coefficients in each case was then taken as the lowest number of coefficients that provided a score that was at maximum 0.01 lower than the maximum score in each case. Prior to calculating the score, the spike reconstruction and sorting accuracies were interpolated with cubic spline interpolation.

In the first part of the analysis, we selected the channel corresponding to the electrode site 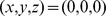 in each of the test recordings (high, medium and low SNR) and quantified performance in terms of the measures described above for all combinations of spike detectors, architectures and compression bases.

In the second part of the analysis, we focused on the *fixed 2* basis and estimated performance in spike sorting and reconstruction at all electrode sites for each test recording, using the ABS detector with architecture 3 and the NEO detector with architecture 1. These detector-architecture combinations were chosen due to their simplicity and their good performances according to the first part of the analysis. We also included NEO detection with architecture 0 (no compression) as a reference.

Figure 3 illustrates the procedure of evaluating performance when compressing spikes recorded at electrode site 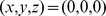 in the high SNR recording for ABS detection, the *fixed 2* basis and architecture 3. The spikes were aligned at the ABS detection threshold to simulate spike detection jitter, aligned at the maximum absolute value of the detected peak/valley, compressed using 8 compression coefficients of the *fixed 2* basis, reconstructed and sorted with PCA and K-means. The alignment was set to have the detected peak aligned with the peak of the first compression basis waveform. The upper panel shows detected and reconstructed spike waveforms (indicates 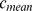 and the lower panel shows the first three PCA features plotted against each other for reconstructed spikes, color-coded according to their true and assigned (by K-means) identities (indicates 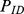).

### 0.8 Computational Complexity on the Implant

To evaluate the system configurations considered in the second part of the analysis in terms of computational complexity, we used similar complexity measures as those employed in [23], where one operation was defined as a one-bit addition. Subtraction was assumed to involve the same number of operations as addition and multiplication and division were assumed to involve ten times as many operations as addition. We assumed a wordlength of 10 bits and a sampling rate of 25 kHz, both of which are within reasonable limits for successful spike detection and spike sorting [6]. Computational complexity was only considered for the cases studied in the second part of the performance estimation (see previous section).

## Results and Discussion

### 0.9 First Part: All Architectures, Detectors and Compression Bases

**Figure 5:**
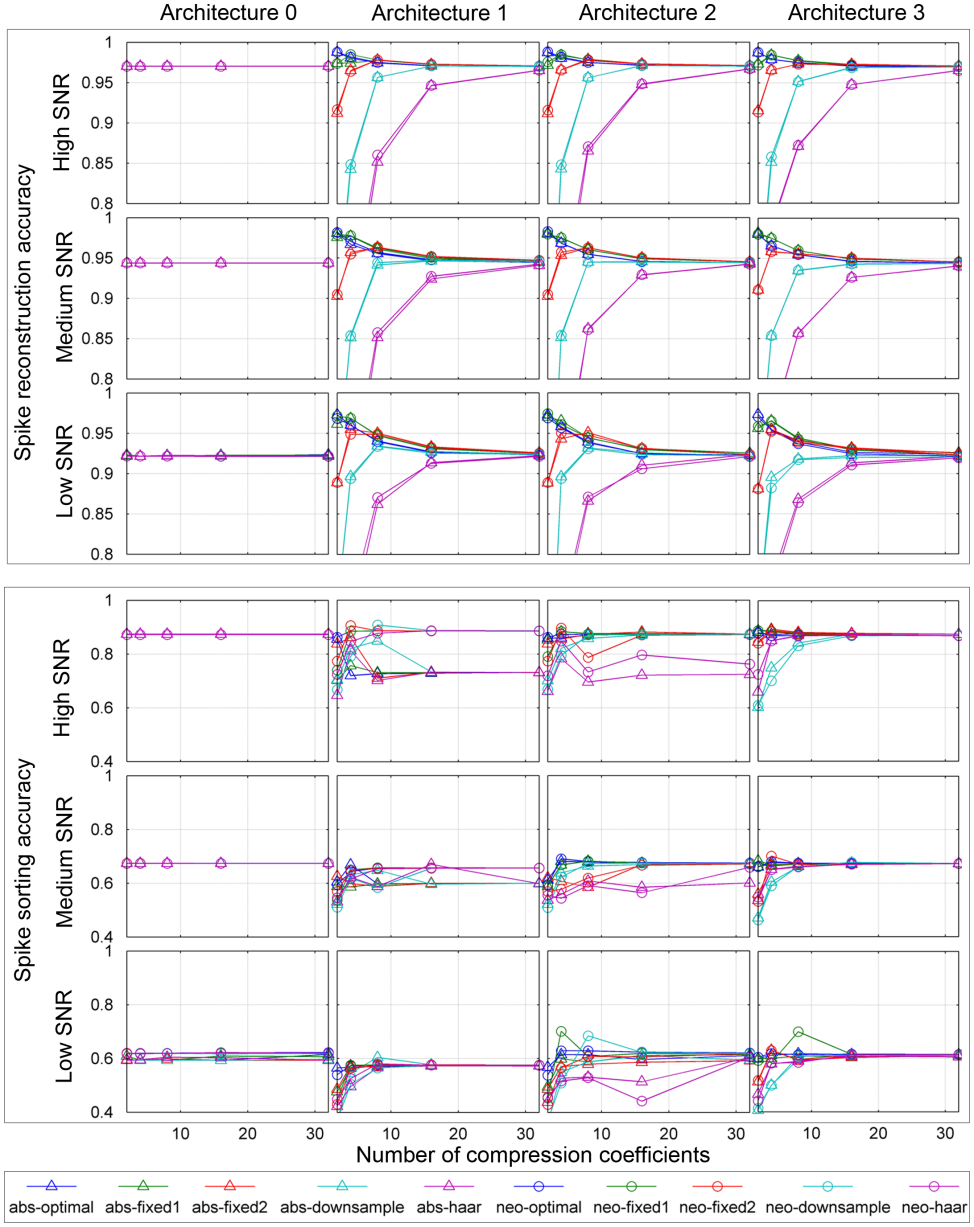
Spike reconstruction (upper panel) and spike sorting (lower panel) as functions of the number of compression coefficients at high, medium and low signal-to-noise ratio (SNR). The columns correspond to the architecture studied (architecture 0 to architecture 3) and each diagram shows the accuracy for each combination of spike detectors and compression bases.

Figure 5 shows spike reconstruction and sorting accuracies as functions of the number of compression coefficients for all system configurations at high, medium and low SNR (first step of performance estimation procedure, see Section 0.7) for the electrode position 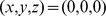.

Spike reconstruction accuracy did not vary noticeably with the choice of architecture, but it did vary significantly with SNR and the choice of compression basis. For a given compression basis, the choice of spike detector did not seem to influence spike reconstruction accuracy. The highest reconstruction accuracy was consistently obtained with the *optimal* and *fixed 1* bases, which was not surprising given that those bases were directly mathematically related to the spike waveforms being compressed. The *fixed 2* basis performed slightly worse at a low number of compression coefficients, or by an accuracy of approximately 0.025 at four coefficients. At eight coefficients, there was no visible difference between the *fixed 2* and the other two SVD based bases. This indicates that in terms of spike reconstruction, the *fixed 2* compression basis is a feasible choice of basis. The *downsampling* and *Haar* bases consistently required a larger number of compression coefficients (16 and 32 respectively) to achieve similar spike reconstruction accuracy as the other three bases. This is also in agreement with our expectation since the most significant transform coefficients of those two bases are not necessarily concentrated at the lower end of the coefficient spectra, as is the case with the SVD based bases, and in order to minimize the computational effort put on selecting compression coefficients, the first 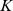 coefficients were selected. For the *optimal*, *fixed 1* and *fixed 2* bases, the reconstruction accuracy was in fact higher than that of the reference case (architecture 0, no compression) and was lowered when adding compression coefficients the decrease being the most significant for the *optimal* basis. This is explained by the fact that reconstruction accuracy was measured by the correlation between reconstructed waveforms and the true mean (noiseless) waveform in each case. Thus, adding compression coefficients added noise to the reconstructed waveform, thereby decreasing the measured reconstruction accuracy.

Spike sorting accuracy was sensitive to all variables, i.e. the choice of architecture, spike detector, compression basis and number of compression coefficients. The sensitivity to the choice of architecture and spike detector is explained by how those components differ in terms of spike alignment, which is known to be important for the performance in spike sorting [2].

The choice of compression basis and the number of compression coefficients did not seem to have as much impact on spike sorting accuracy as on spike reconstruction accuracy, indicating that although distorted by the compression, spikes can still be sorted successfully, given the appropriate choice of architecture, spike detector and spike sorting algorithm. In general, the SVD based bases (*optimal*, *fixed 1* and *fixed 2*) required a smaller number of compression coefficients (two to four) to achieve maximal spike sorting accuracy. The *downsampling* and *Haar* bases required approximately 8 compression coefficients for maximum performance.

For architecture 1, spike sorting accuracy was mostly influenced by the choice of spike detector, the NEO detector generally resulting in better performance. The difference was as high as 20% at high SNR, but insignificant at low SNR. This indicates that the use of the NEO detector reduces the need for a separate spike alignment step prior to spike sorting. However, the decreasing difference with decreased SNR indicates that this attractive property of the NEO detector only applies at high SNR conditions.

By moving to architecture 2, and thereby introducing alignment after externally reconstructing the spikes, the influence of the choice of spike detector became less obvious. For instance, the Haar basis consistently performed the worst (for both detectors) but for all other bases, the choice of detector did not seem to influence spike sorting accuracy. For the *fixed 2* basis, the dependence of the number of compression coefficients and spike detector was somewhat inconsistent. At high and low SNR and with the ABS detector, it performed similarly to the other two SVD based bases and the downsampling basis, but at medium SNR, it required as many as 16 compression coefficients to achieve maximum performance.

For architecture 3, where spike alignment is introduced prior to spike compression, the performances became more consistent and the choices of spike detector and compression basis became less significant. The independence of spike detector choice is explained by the fact that after alignment, the spikes extracted by the two detectors are essentially identical.

Note that for architecture 3, the spike reconstruction accuracy for the *downsampling* basis is generally higher than that of the *Haar* basis, but the opposite is true for spike sorting accuracy. Although this may seem counterintuitive, it can be explained in terms of the characteristic nature of the two transforms. In downsampling, compression is obtained by discarding waveform samples and in reconstruction, the remaining samples are interpolated and smoothed, which effectively ''erases'' detailed waveform landmarks that may prove essential for spike sorting, where minor differences between spikes coming from different neurons are essential for performance. However, the waveforms maintain their overall shapes, resulting in high correlation with the true mean spike waveforms and therefore a high measured reconstruction accuracy. In the wavelet transform, on the other hand, the waveform is distorted by the compression, making it significantly different from the mean spike waveform, but the details may be conserved in the transform coefficients, resulting in less impact on spike sorting accuracy.

**Figure 6:**
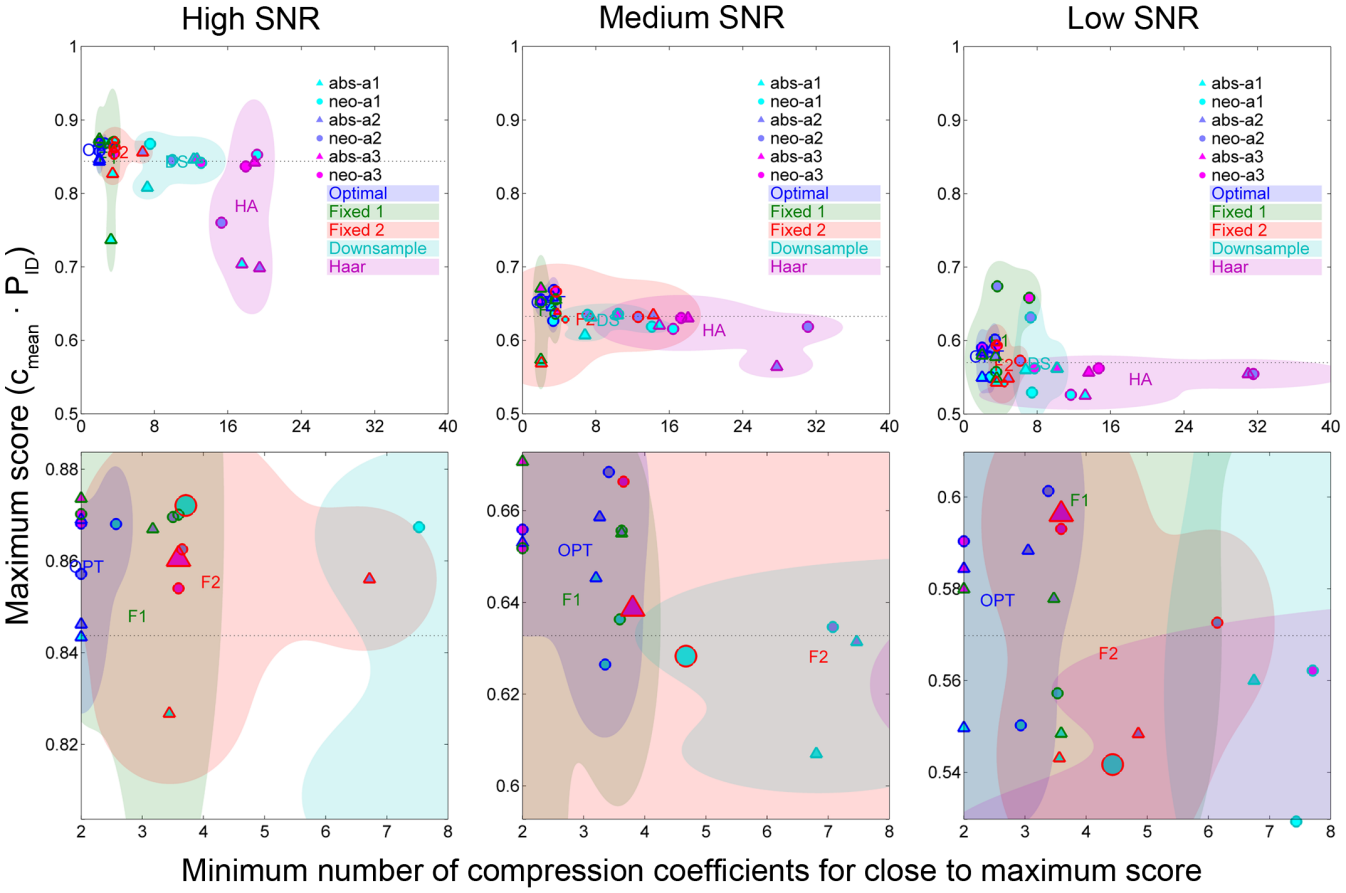
Performance scores and minimum number of compression coefficients needed for all system configurations analyzed. The ''clouds'' indicate the area covered by the different compression bases and the icons within the clouds represent the different combinations of spike detector and system architecture for each basis. The upper panels show the entire range of compression coefficients and scores and all configurations. The lower panels focus on the area around the cases where the *fixed 2* compression basis is used.

The upper part of Figure 6 shows the maximum achievable scores and the minimum number of compression coefficients for all system configurations. The score obtained for uncompressed spikes is shown by the dotted line for comparison. Each ''cloud'' indicates the range of performances covered by the different combinations of system architecture and spike detector for the different compression bases. For instance, at high SNR, the *Haar* basis (violet cloud) generally required the largest number of compression coefficients, and the selection of architecture and spike detector was significant for the maximum achievable performance at high SNR. Some of the configurations (those including architecture 3) reached similar performance as when no compression was performed, but at the cost of transmitting more than 16 compression coefficients. The *fixed 2* basis (red cloud), on the other hand, consistently required a significantly lower number of compression coefficients and the maximum achievable score was less sensitive to the choice of architecture and spike detector.

The lower part of Figure 6 shows a magnified part of the upper panels, focusing on the *fixed 2* basis. The two cases of special interest are highlighted with enlarged symbols, i.e. NEO detection with architecture 1 and ABS detection with architecture 3. At high SNR, both configurations performed better than the reference case (no compression), which can be explained by the noise reduction that is introduced with the compression. As SNR decreases, NEO detection with architecture 1 fell below the reference case in performance, while the performance of ABS detection with architecture 3 was sustained. ABS detection with architecture 3 required the same or smaller number of compression coefficients as/than NEO with architecture 1. Note that the score axis is kept at a fixed scale for the different SNRs, but is centered at the score of the reference case (no compression). These observations are the reason for focusing on these cases in more detail in the second part of the analysis.

### 0.10 Second Part: NEO and Fixed 2 with Architecture 1 and ABS and Fixed 2 with Architecture 3

**Figure 7:**
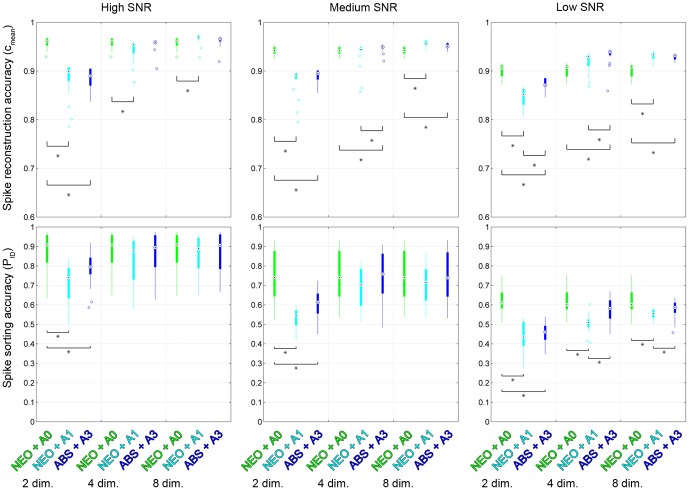
Spike reconstruction accuracy (upper row) and spike sorting accuracy (lower row) for NEO detection with architecture 0 (no compression, reference case), NEO detection with architecture 1 and compression with the *fixed 2* basis and ABS detection with architecture 3 and compression with the *fixed 2* basis at high, medium and low SNR. The distributions describe the performance across all nineteen electrode sites in each case. Median comparison intervals (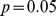) are marked with triangles and significantly different cases are marked with a star and a bracket.

Figure 7 shows the distributions of accuracies in spike reconstruction and spike sorting for NEO detection with architecture 0 (no compression, reference case), NEO detection with architecture 1 and compression with the *fixed 2* basis and ABS detection with architecture 3 and compression with the *fixed 2* basis at high, medium and low SNR, over the recording electrode sites. Significant difference (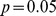) between cases is indicated with stars and brackets. As discussed previously, these cases were selected for further investigation due to their good performances and requirement for few compression coefficients according to the first part of the analysis (see previous section).

In general, a decreased SNR led to a decrease in spike reconstruction accuracy. At a given noise level, spike reconstruction accuracy was generally enhanced by compression when the number of compression coefficients was at least four. This is due to the noise reduction introduced by transform coding with SVD based compression bases. Note however, that the spike detection performance generally decreases with decreased SNR [6], [23] so this is not an indication that low SNR is beneficial – but rather that given a low SNR, compression is an efficient way of reducing noise in the detected spike waveforms.

As expected, increasing the number of compression coefficients increased the reconstruction accuracy for both configurations. Both configurations required at least four compression coefficients for the spike reconstruction accuracy to be equal to or higher than that of the reference configuration. In the few cases (medium SNR – 4 dimensions, low SNR – 2 dimensions, low SNR – 4 dimensions) where there was a significant difference between the spike reconstruction accuracies of the two configurations involving compression, ABS detection with architecture 3 performed better.

Also as expected, spike sorting accuracy generally decreased with a decreased SNR and tended to decrease with a decreased number of compression coefficients. At two compression coefficients, both compression configurations provided spike sorting accuracy that was significantly lower than that obtained in the reference case. At four and eight coefficients at high and medium SNR, all configurations performed equally, but at low SNR NEO detection with architecture 1 provided significantly lower performance than both the reference architecture and ABS detection with architecture 3. Even at low SNR, ABS detection with architecture 3 was close in performance to the reference case at four and eight compression coefficients.

The computational complexity at the implant for NEO with architecture 1 and ABS with architecture 3 was 5.5 MOPS/spike/dimension and 0.51 MOPS/spike/dimension respectively. The large (factor 10) difference between the complexities was due to the increased complexity introduced by performing spike detection with NEO.

## Conclusions

In the present paper, we have presented our analysis of various combinations of spike detectors, compression architectures and compression bases with regard to performance in spike reconstruction and spike sorting. The analysis has been carried out at various signal to noise ratios and a reference case was included in which no compression was performed. Due to the inherent constraints of wireless BMIs, which require the minimization of both computational cost and data rate, we have focused on non-adaptive implant designs, i.e. designs where compression is performed with fixed compression bases and a fixed set of compression coefficients is transmitted. This relieves the implant of the computational burden of finding and maintaining an optimal compression basis. For the same reasons, we have not considered methods for dealing with variations in spike shape caused by overlapping spikes, bursting or electrode drift. For the bursting and electrode drift cases however, we reason that the changes in spike shape should not significantly influence performance from the point of view of compression with a fixed generic compression basis. This reasoning is assumed to hold as long as the variation in spike shape caused by bursting or electrode drift is within the range of spike shapes covered by the spike library used to derive the fixed generic compression basis.

The first part of the analysis indicates that given the appropriate choice of spike detector and system architecture, spike waveform compression with a fixed basis derived by singular value decomposition (SVD) of a set of empirically obtained spike waveforms is beneficial compared to using a generic basis such as the Haar wavelet basis or downsampling when no measures are taken to optimize coefficient selection. Also, the *fixed 2* basis provides close to optimal performance, given that at least four compression coefficients are transmitted. This is due to the high degree of sparsification (dimensionality reduction) and the compact distribution of significant coefficients in the lower end of the coefficient spectrum, which facilitates a high degree of compression without the need for searching for the most significant coefficients (the first 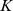 coefficients are transmitted). In order to maximize performance in spike sorting, spike alignment is needed and can be established either by waveform-shifting or by spike detection with the nonlinear energy operator (NEO). However, this benefit of the NEO detector is only present at high SNR and the NEO detector is an order of magnitude more complex than the absolute value threshold detector (ABS). From the point of view of spike reconstruction, the choice of architecture and spike detector are of minor importance, while the choice of a compression basis and dimensionality are critical – the SVD based bases being the most beneficial in the absence of optimization procedures for compression coefficient selection for the other bases.

The second part of this paper focuses on compression with the *fixed 2* basis. Two cases, selected based on the comparison in the first part, and a reference case in which no compression is performed, are included. The cases of interest are the NEO detector with architecture 1 (spike alignment introduced by the detector) and the ABS detector with architecture 3 (spikes aligned on the detected peak prior to compression). The results of this part show that at least four coefficients need to be transmitted in order for the compression not to significantly degrade performance. Due to the noise reduction introduced by transform coding with an SVD based basis, spike reconstruction can be improved by compression. At high and medium SNR, both compression architectures perform equally well, but at low SNR, NEO detection with architecture 1 falls behind.

Considering the results of our analysis and the challenges involved in designing low-power multichannel wireless BMIs, we propose a spike compression architecture that consists of absolute value threshold detection, spike alignment at the implant and compression with a fixed basis that is derived by SVD from a large assembly of empirically found spike waveforms, allowing a straight-forward selection of compression coefficients to transmit. Such a configuration has been shown to provide spike reconstruction and sorting accuracies that differ insignificantly from those obtained when no compression is performed, given that at least four compression basis coefficients are transmitted per detected spike waveform.

Transmitting four compression coefficients per detected spike waveform and assuming 10 bits per sample results in a data rate of 40 bits per transmitted spike waveform, or a factor of 16 times less than when transmitting the uncompressed spike waveforms (assuming 25 kHz sampling rate and 2.5 ms spike duration, i.e. 64 samples). In order to simplify these comparisons, we do not consider overhead data such as timestamps and channel IDs. Assuming a mean of four neurons per recording channel and a mean firing rate of 10 spikes per second per neuron, this corresponds to a mean total data rate of 1.6 kbps, or a 99.8% reduction compared to when transmitting the raw acquired data. Assuming a wireless link capacity of 1 Mbps, this would allow the transmission of spike data from 625 recording channels, or a factor of 156 times more than when transmitting the raw neural data (four channels maximum).

Due to the variability in implementation and validation methods employed in publications within the field, direct comparisons of the previously reported approaches is not straight-forward. However, based on the comparison performed in the present study, we conclude that our proposed architecture using a fixed generic compression basis derived from spike data is at least competitive in terms of both compression ratio and computational complexity. This conclusion is based on the following: 1) Performance in spike reconstruction and sorting is not influenced by the compression when transmitting as few as four compression coefficients, resulting in an excellent compression ratio. 2) After reconstruction, the spike waveforms are available at the receiver side. 3) The use of a low-complexity spike detector in the time domain prior to transform coding lowers the frequency of transform coding operations. 4) The use of a fixed compression basis derived from experimentally obtained spike waveforms eliminates the need for training the compression architecture and computing transform coefficients that would be discarded in a thresholding procedure carried out in the transform domain.

## References

[1] Lebedev MA, Nicolelis MaL (2006) Brain-machine interfaces: past, present and future. Trends in neurosciences 29: 536–46. 16859758 10.1016/j.tins.2006.07.004

[2] Mitra P, Bokil H (2008) Observed Brain Dynamics. Oxford University Press, USA, 408 pp. Available: http://www.amazon.com/Observed-Brain-Dynamics-Partha-Mitra/dp/0195178084.

[3] Abeles M, Goldstein M (1977) Multispike train analysis. Proceedings of the IEEE 65: 762–773.

[4] Lewicki MS (1998) A review of methods for spike sorting: the detection and classification of neural action potentials. Network (Bristol, England) 9: R53–78. 10221571

[5] Einevoll GT, Franke F, Hagen E, Pouzat C, Harris KD (2012) Towards reliable spike-train recordings from thousands of neurons with multielectrodes. Current opinion in neurobiology 22: 11–7. 22023727 10.1016/j.conb.2011.10.001 PMC3314330

[6] Thorbergsson PT, Garwicz M, Schouenborg J, Johansson AJ (2012) Minimizing data transfer with sustained performance in wireless brain-machine interfaces. Journal of neural engineering 9: 036005. 22523005 10.1088/1741-2560/9/3/036005

[7] Maynard EM, Nordhausen CT, Normann RA (1997) The Utah intracortical Electrode Array: a recording structure for potential brain-computer interfaces. Electroencephalography and clinical neurophysiology 102: 228–39. 9129578 10.1016/s0013-4694(96)95176-0

[8] Fee MS, Mitra PP, Kleinfeld D (1996) Automatic sorting of multiple unit neuronal signals in the presence of anisotropic and non-Gaussian variability. Journal of neuroscience methods 69: 175–88. 8946321 10.1016/S0165-0270(96)00050-7

[9] Thorbergsson PT, Garwicz M, Schouenborg J, Johansson AJ (2010) Statistical modelling of spike libraries for simulation of extracellular recordings in the cerebellum. Conference Proceedings of the International Conference of IEEE Engineering in Medicine and Biology Society 2010: 4250–4253. 10.1109/IEMBS.2010.5627177 21096640

[10] Donoho D (2006) Compressed sensing. Information Theory, IEEE Transactions on 52: 1289–1306.

[11] Narasimhan S, Tabib-Azar M, Chiel HJ, Bhunia S (2007) Neural Data Compression with Wavelet Transform: A Vocabulary Based Approach. 2007 3rd International IEEE/EMBS Conference on Neural Engineering: 666–669.

[12] Aghagolzadeh M, Oweiss K (2009) Compressed and distributed sensing of neuronal activity for real time spike train decoding. IEEE transactions on neural systems and rehabilitation engineering: a publication of the IEEE Engineering in Medicine and Biology Society 17: 116–27. 10.1109/TNSRE.2009.2012711 PMC2782557 19193517

[13] Charbiwala Z, Karkare V, Gibson S, Markovic D, Srivastava MB (2011) Compressive Sensing of Neural Action Potentials Using a Learned Union of Supports. 2011 International Conference on Body Sensor Networks: 53–58.

[14] Hosseini-Nejad H, Jannesari A, Sodagar AM (2013) Data Compression in BrainMachine/Computer Interfaces Based on the WalshHadamard Transform. IEEE Transactions on Biomedical Circuits and Systems PP. 10.1109/TBCAS.2013.2258669 24681926

[15] Jörntell H, Ekerot CF (2002) Reciprocal bidirectional plasticity of parallel fiber receptive fields in cerebellar Purkinje cells and their afferent interneurons. Neuron 34: 797–806. 12062025 10.1016/s0896-6273(02)00713-4

[16] Thorbergsson PT, Jorntell H, Bengtsson F, Garwicz M, Schouenborg J, et al (2009) Spike library based simulator for extracellular single unit neuronal signals. Conference proceedings: Annual International Conference of the IEEE Engineering in Medicine and Biology Society IEEE Engineering in Medicine and Biology Society Conference 2009: 6998–7001. 10.1109/IEMBS.2009.5333847 19964726

[17] Thorbergsson PT, Garwicz M, Schouenborg J, Johansson AJ (2012) Computationally efficient simulation of extracellular recordings with multielectrode arrays. Journal of Neuroscience Methods 211: 133–144. 22960053 10.1016/j.jneumeth.2012.08.011

[18] Lempka SF, Johnson MD, Moffitt MA, Otto KJ, Kipke DR, et al (2011) Theoretical analysis of intracortical microelectrode recordings. Journal of neural engineering 8: 045006. 21775783 10.1088/1741-2560/8/4/045006 PMC3196618

[19] Buzsáki G (2004) Large-scale recording of neuronal ensembles. Nature neuroscience 7: 446–51. 15114356 10.1038/nn1233

[20] Heeger D (2000) Poisson model of spike generation. Handout, University of Standford: 1–13.

[21] Kim KH, Kim SJ (2000) Neural spike sorting under nearly 0-dB signal-to-noise ratio using nonlinear energy operator and artificial neural-network classifier. IEEE transactions on bio-medical engineering 47: 1406–11. 11059176 10.1109/10.871415

[22] Quiroga RQ, Nadasdy Z, Ben-Shaul Y (2004) Unsupervised spike detection and sorting with wavelets and superparamagnetic clustering. Neural computation 16: 1661–87. 15228749 10.1162/089976604774201631

[23] Gibson S, Judy JW, Marković D (2010) Technology-aware algorithm design for neural spike detection, feature extraction, and dimensionality reduction. IEEE transactions on neural systems and rehabilitation engineering: a publication of the IEEE Engineering in Medicine and Biology Society 18: 469–78. 10.1109/TNSRE.2010.2051683 20525534

[24] Obeid I, Wolf PD (2004) Evaluation of spike-detection algorithms for a brain-machine interface application. IEEE transactions on bio-medical engineering 51: 905–11. 15188857 10.1109/TBME.2004.826683

[25] Mukhopadhyay S, Ray GC (1998) A new interpretation of nonlinear energy operator and its efficacy in spike detection. IEEE transactions on bio-medical engineering 45: 180–7. 9473841 10.1109/10.661266

[26] Meyer C (2000) Matrix analysis and applied linear algebra. SIAM, Philadelphia 1: 1829–1841.

[27] Mitra SK (2001) Digital Signal Processing: A Computer-Based Approach. McGraw-Hill/Irwin, 866 pp.

[28] Duda RO, Hart PE, Stork DG (2001) Pattern classification. New York, NY: Wiley, 2nd edition, 654 pp. Available: http://books.google.com/books?id=YoxQAAAAMAAJ&pgis=1.

[29] Pavlov A, Makarov VA, Makarova I, Panetsos F (2007) Sorting of neural spikes: When wavelet based methods outperform principal component analysis. Natural Computing 6: 269–281.

[30] Jackson A, Fetz EE (2007) Compact movable microwire array for long-term chronic unit recording in cerebral cortex of primates. Journal of neurophysiology 98: 3109–18. 17855584 10.1152/jn.00569.2007

